# Nanoparticles Based on Silver Chloride and Bambusuril[6] for the Fine-Tuning of Biological Activity

**DOI:** 10.3390/ijms242216126

**Published:** 2023-11-09

**Authors:** Pana Turebayeva, Venera Luchsheva, Dmitriy Fedorishin, Rakhmetulla Yerkassov, Abdigali Bakibaev, Saltanat Bolysbekova, Tokzhan Tugambayeva, Samal Sergazina, Nurgul Nurmukhanbetova

**Affiliations:** 1 Department of Chemistry, L.N. Gumilyov Eurasian National University, 010000 Astana, Kazakhstan; pana90@mail.ru (P.T.); erkass@mail.ru (R.Y.); 2 Faculty of Chemistry, National Research Tomsk State University, 634050 Tomsk, Russia; kuscherbaeva_venera@mail.ru (V.L.); strix187@yandex.ru (D.F.); 3 Higher School of Scientific Research, Astana International University, 010000 Astana, Kazakhstan; bolysbekova.s@mail.ru; 4 Department of Chemistry and Chemical Technologies, Toraighyrov University, 140008 Pavlodar, Kazakhstan; togzhan_1963@mail.ru; 5 Department of Chemistry and Biotechnology, Sh. Ualikhanov University, 020000 Kokshetau, Kazakhstan; samal_sergazina@mail.ru (S.S.); nn_nurgul@mail.ru (N.N.)

**Keywords:** bambusuril[6], silver chloride nanoparticles, MTT-test, thermal analysis, supramolecular chemistry, antibacterial activity

## Abstract

The prevalence of numerous infectious diseases has emerged as a grave concern within the realm of healthcare. Currently, the issue of antibiotic resistance is compelling scientists to explore novel treatment approaches. To combat these infectious diseases, various treatment methods have been developed, harnessing cutting-edge disinfecting nanomaterials. Among the range of metallic nanoparticles employed in medicine, silver nanoparticles (AgNPs) stand out as both highly popular and well-suited for the task. They find extensive utility in cancer diagnosis and therapies and as effective antibacterial agents. The interaction between silver and bacterial cells induces significant structural and morphological alterations, ultimately leading to cell demise. In this study, nanoparticles based on silver and bambusuril[6] (BU[6]) were developed for the first time. These NPs can be used for different biomedical purposes. A simple, single-step, and effective synthesis method was employed to produce bambusuril[6]-protected silver chloride nanoparticles (BU[6]-Ag/AgCl NPs) through the complexation of BU[6] with silver nitrate. The NPs were characterized using X-ray phase analysis (XPS), infrared spectroscopy (IR), thermogravimetric analysis (TGA), scanning electron microscopy (SEM) and energy-dispersive X-ray spectroscopy (EDS). When the SEM images were examined, it was seen that the synthesized BU[6]-Ag/AgCl NPs were distributed with homogeneous sizes, and the synthesized NPs were mostly spherical and cubic. The EDS spectra of BU[6]-Ag/AgCl NPs demonstrated the presence of Ag, Cl, and all expected elements. BU[6]-Ag/AgCl NPs showed high antibacterial activity against both *E. coli* and *S. aureus* bacteria.

## 1. Introduction

Currently, metallic nanoparticles based on silver (Ag), gold (Au), and copper (Cu) are widely employed in various applications such as biosensors, catalysis, targeted drug delivery, and antibacterial therapies. Among these nanoparticles, silver nanoparticles AgNPs have attracted significant attention due to their exceptional antibacterial activity against diverse microorganisms, including bacteria, viruses, and fungi [1,2,3,4,5,6].

Various reagents have been used to synthesize AgNPs. Green synthesis of silver–silver chloride nanoparticles (Ag–AgCl NPs) has been proposed as a simple, easy, eco-friendly and cost-effective method. Ag/AgCl NPs synthesized from *Azadirachta indica* lalex are currently known to be active against fluconazole-resistant *Candida tropicalis* [7]. There is a known easy and green synthesis method for carrageenan-coated silver NPs. The nanoparticles have antimicrobial activity for *E. coli* and *S. aureus* bacteria [8]. The leaf extract of Sasa borealis is a source for the reduction of silver nitrate into Ag-AgCl NPs. Phytochemicals of the leaves act as both reducing and stabilizing agents. Sasa borealis Ag-AgCl NPs exhibit significant antibacterial activity against Gram-positive and Gram-negative pathogens and anticancer activity against AGS (gastric adenocarcinoma) cells [9]. Novel Ag-AgCl NPs were developed from the bacteria *Shewanella* sp. Arc9-LZ, which were isolated from the deep sea of the Arctic Ocean [10]. These nanoparticles have negative effects on the breast cancer cell line MCF-7 [11,12].

The unique properties of AgNPs are directly influenced by their sizes and shapes, with AgNPs typically consisting of 20 to 15,000 silver atoms and exhibiting sizes ranging from 1 to 100 nm [13,14]. Moreover, due to the large surface area to volume ratio, NPs exhibit remarkable antimicrobial activity, even at low concentrations [15]. AgNPs possess the ability to combat both aerobic and anaerobic microorganisms, making them effective agents for microbial eradication [16]. The remarkable antibacterial properties of AgNPs stem from their ability to interact with the disulfide (S-S) bonds found in metabolic enzymes, disrupting the cellular integrity and impairing respiratory processes [17]. Upon contact with bacteria, these nanoparticles adhere to the cell wall and membrane, where they exhibit dual modes of action. Some AgNPs penetrate the interior, interacting with phosphate-containing compounds such as DNA and RNA, whereas others bind to sulfur-containing proteins on the membrane [18].

Interestingly, AgNPs, in addition to antibacterial activity, exhibit anticancer properties against various cancer cell lines, including breast cancer cells (MCF-7) [19], colon cancer cells (HCT116) [20], prostate cancer cells [21], and lung carcinoma cells. The anticancer activity of AgNPs is attributed to their ability to induce cell death in mammalian cells. AgNPs accumulate within endosomes upon entering the body and subsequently fuse with lysosomes. Within the acidic environment of lysosomes, AgNPs release Ag^+^ ions at an increased rate. These reactive ions disrupt cellular homeostasis and, depending on the specific characteristics of the target cells, can trigger apoptotic cell death [22]. This mechanism of action, often referred to as the “Trojan horse” mechanism, showcases the cytotoxic properties of AgNPs post cellular uptake [23,24]. Additionally, AgNPs have shown potential for enhancing the effectiveness of combined radiation and chemotherapy treatments [25].

Supramolecular compounds have gained significant recognition beyond nanoparticles. Supramolecular chemistry aims to utilize non-covalent interactions to construct intricate chemical systems [26,27,28,29,30]. Most commonly, the interacting species are held together by hydrogen bonds. The definition excludes compounds formed by electrostatic interactions, which are referred to as ion pairs. Among the various supramolecular architectures, macrocycles have emerged as highly versatile entities due to their inherent cavities that are capable of hosting guest molecules [31,32,33]. The expansion and change in size of the ring cavity and the selective complexation of macrocyclic compounds with inorganic and organic small molecules and metal ions have brought new attention to macrocyclic chemistry. Chemists have extensively researched macrocycles and their derivatives to achieve structure-specific and highly selective recognition properties, which provide opportunities for exploring advanced applications in sensing, transport, catalysis, and drug/gene delivery. Nitrogen-containing heterocycles are structures that are widely found in natural products and pharmaceutical molecules. Compounds containing such structures often have unique physiological and pharmacological properties. Macrocycles based on Bambusuril[n] (BU[n]) were discovered relatively recently by the scientists Jan Svec and Vladimir Sindelar in 2010. BU[n] represents a new class of macrocyclic compounds consisting of n-2,4-substituted glycoluril units connected by a single row of methylene bridges. These macrocycles combine the structural features of both cucurbituril[n] and hemicucurbituril[n] [34]. At a height of 12.7 A, BU[6] has a significantly deeper cavity than cucurbit[n]urils, whose height is 9.1 A. BU[6] has a high affinity for negatively infected molecules and ions [35,36,37]. Recent studies focused on the chemistry of bambusuril and its use as an effective and selective adsorbent for anions [38,39]. Recent discoveries have revealed that bambusuril can selectively bind to [Au(CN)_2_]^−^ ions [40], suggesting their potential application in the gold mining industry. Additionally, it has been found that semithiobambusuril, featuring terminal thiocarbonyl groups, has a fascinating ability to form highly stable and well-ordered monolayers on gold surfaces. The attachment of bambusuril molecules to gold surfaces induces significant conformational changes.

The chemistry of bambusuril is actively developing; however, there are still many questions regarding its supramolecular properties. It is known that bambusuril possesses “host–guest” properties [40,41], but to date, the suitability of BU[6] for the synthesis and stabilization of metallic nanoparticles, particularly AgNPs, has not been investigated. Previously, AgNPs stabilized with cucurbituril[7] were obtained in an aqueous medium in the presence of NaOH at room temperature [42].

In our previous studies, we developed a biomaterial based on porous titanium nickelide and bambusuril[6] [43,44]. By employing a supramolecular methodology, a complex that combines bambusuril[6] and benzalkonium chloride was successfully synthesized. The ongoing advancement of supramolecular systems based on bambusuril[6] and therapeutic agents shows great promise for the creation of novel materials with the ability to release drugs over an extended period under the influence of various factors.

The main concept of this work is to create new BU[6]-protected AgNPs. In this work, we obtained NPs by a simple method using the reaction of silver nitrate with bambusuril[6] in DMSO/CHCl_3_ at room temperature.

## 2. Results and Discussion

**Scheme 1:**
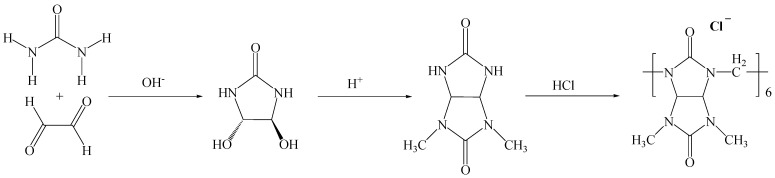
Synthesis of bambusuril[6].

In the first stage of our work, BU[6] was synthesized according to the traditional approach [35] of 2,4-dimethylglycoluryl and the acid-catalyzed Mannich-type condensation of disubstituted glycoluril with paraformaldehyde in a solvent at reflux (Scheme 1).

**Figure 1:**
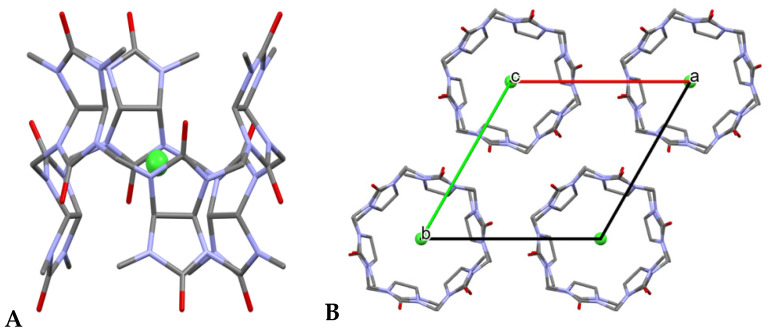
The X-ray crystal structure of BU[6] (**A**) cross-section of BU[6], (**B**) cell parameters. The interior cavity of bambusuril[6] accommodates Cl^−^.

The structure of the obtained bambusuril was confirmed through NMR and X-ray crystallography. The X-ray structural analysis (Figure 1) of bambusuril[6] revealed that the macrocycle contains six structural units. Bambusuril[6] adopts a monoclinic symmetry, and its crystalline lattice belongs to the space group P-6. The cell parameters were determined as follows: a—12.2731 Å (10), b—12.2731 Å (10), c—31.569 Å (2); *α* = 90, *β* = 90, *γ* = 120. Notably, the interior cavity of bambusuril[6] accommodates the Cl^−^ ion. The presence of a templating anion is essential for the preferential formation of BU[6] over BU[4].

**Figure 2:**
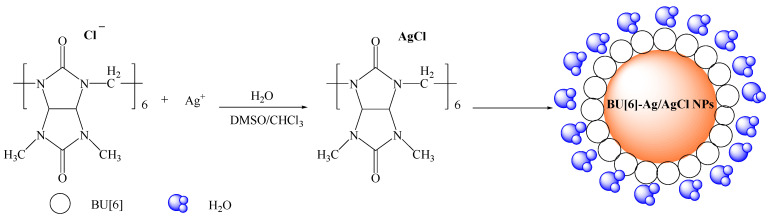
The scheme shows the synthesis of BU[6]-Ag/AgCl NPs.

In the experiment, we dissolved bambusuril[6] in DMSO/CHCl_3_ and added AgNO_3_ and water, and the addition of water led to the formation of a brownish-gray solution, indicating the formation of AgNPs (Figure 2).

Under certain conditions, it is anticipated that BU[6] would preferentially assist with the adsorption, reduction, and growth of metal, which would result in the formation of nanoscale structures. The diverse polar carbonyl portals in BU[6] contribute significantly to the reduction of Ag^+^ ions via cumulative negative surface charges, resulting in the formation of AgNPs. Bambusuril[6] contains Cl^−^ inside its cavity (Figure 1); therefore, Ag^0^ binds to Cl^−^ inside its cavity, forming BU[6]-Ag/AgCl NPs. As the metallic particles condense to the nanoscale, effective stabilization is achieved through either electrostatic or steric mechanisms. BU[6] molecules serve as excellent stabilizers by forming a protective coating on the silver nanoparticle surfaces, counteracting Van der Waals forces that promote particle agglomeration. We propose a possible interaction model in which the carbonylated portals of BU[6] interact with the surfaces of the NPs, similar to the behavior observed with CB[7] [42]. Experimental evidence, supported by an FTIR analysis, demonstrates a notable decrease in the intensity of the characteristic carbonyl peak of BU[6] in the spectrum of the BU[6]-stabilized Ag/AgCl NPs. Additionally, a significant high-frequency shift from 1683 to 1694 cm^−1^ indicates supramolecular interactions between the carbonyl groups and the NPs surfaces.

**Figure 3:**
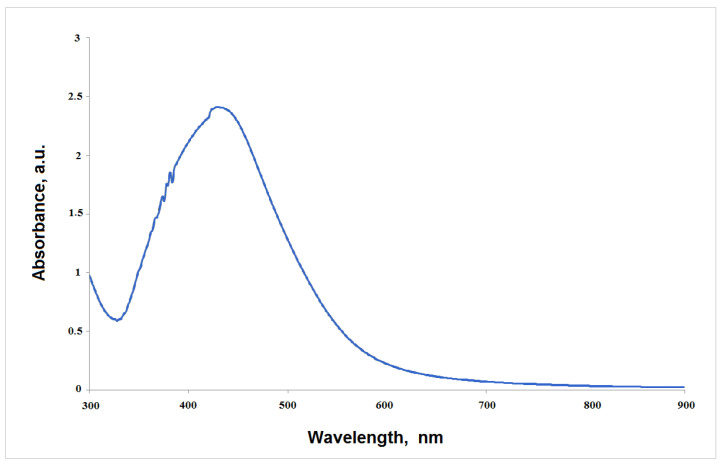
UV-visible spectrophotometry of BU[6]-AgCl NPs.

Further, the formation of AgNPs was confirmed using UV-visible spectrophotometry (Figure 3). BU[6]-Ag/AgCl NPs absorbed light in the visible region due to surface plasmon resonance and produced a singular peak point at 430 nm [45].

The solution of AgNO_3_ in the presence of DMSO/CHCl_3_ did not show the characteristic surface plasmon band, indicating that bambusuril[6] may play a decisive role in the reduction of silver salts to Ag^0^. Our results are in agreement with the literature, as the UV-visible spectra show that silver NPs have similar wavelength characteristics [46,47].

**Figure 4:**
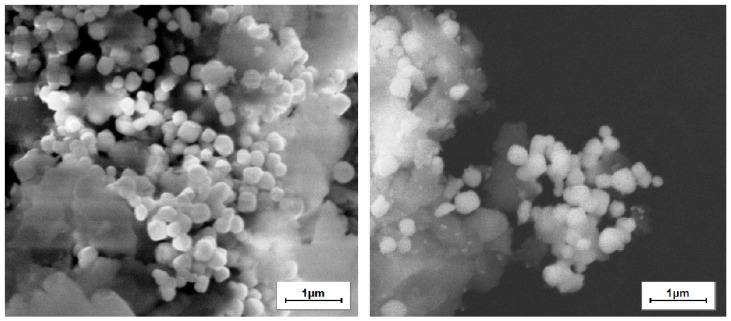
SEM image of synthesized BU[6]-Ag/AgCl NPs.

**Figure 5:**
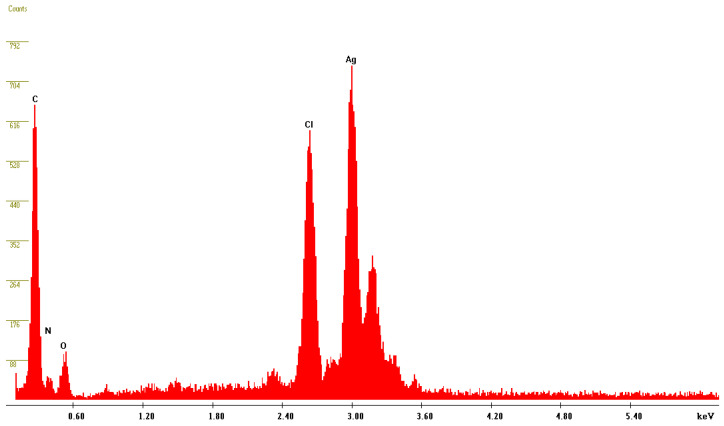
EDS analysis of synthesized BU[6]-Ag/AgCl NPs.

The morphologies of the synthesized particles were examined by SEM, and the SEM BU[6]-Ag/AgCl NPs samples are shown in Figure 4. When the SEM images were examined, it was seen that the synthesized BU[6]-Ag/AgCl NPs were distributed with homogeneous sizes, and the synthesized AgNPs were mostly spherical and cubic. Certain elements found in the BU[6]-Ag/AgCl NPs were determined by the EDS analysis, and the results are given in Figure 5. The EDS spectra of the BU[6]-Ag/AgCl NPs showed the presence of Ag, Cl, and all expected elements. A strong signal at 3 keV revealed the presence of metallic silver in BU[6]-Ag/AgCl NPs [48]. It was also shown that BU[6]-Ag/AgCl NPs did not contain any impurities. The C, O, and N peaks are evidence that bambusuril[6] was used during BU[6]-Ag/AgCl NP synthesis.

**Figure 6:**
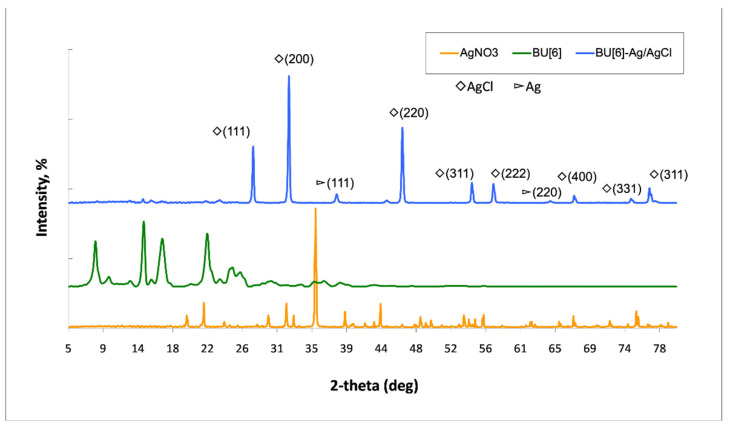
XRD patterns of BU[6], AgNO_3_, BU[6]-Ag/AgCl NPs.

The crystalline nature of the synthesized silver nanoparticles, bambusuril[6], and AgNO_3_ was investigated using X-ray diffraction (XRD). The XRD pattern of BU[6]-Ag/AgCl NPs showed peaks at 27°, 32°, 46°, 54°, 57°, 67°, 74°, and 77° (Figure 6), corresponding to the different orientation planes at 111, 200, 220, 311, 222, 400, 331, and 311 for the AgCl NPs, indicating a face-centered cubic structure of silver crystals (JCPDS card No. 31-1238). Additionally, some lower peaks were seen at 38° and 65° at an angle of 2*θ*, which indicated the cubic phase of Ag NPs (JCPDS no. 65-2871). Thus, the XRD pattern clearly demonstrates that the formed NPs have a crystalline nature. The present findings are in good agreement with previous studies of silver chloride NPs synthesis using arctic Marine Bacterium [10], leaf extract of pineapple peel [12], and *Sasa borealis* [9].

Reflections from the (111), (200), (220), (311), and (222) lattice planes of AgCl NPs can be seen as a series of intense Bragg reflections. All of the reflections are consistent with the crystalline structure of silver chloride with a face-centered cubic symmetry. The high degree of crystallinity of the silver NPs is evident in the intensities of the peak position reflections. The crystallite size of silver NPs was determined using the Debye−Scherrer equation:

$$D=\frac{0.89\lambda }{\beta \cos\theta }$$

where the Cu Kα X-ray wavelength *λ* = 0.154056 nm, *θ* is Bragg’s diffraction angle (° or radian), and *β* (radian) is the full width at half-maximum (FWHM) of the maximum intensity peak °. The results show that the average crystallite size of BU[6]-AgCl NPs is 25.35 nm (Table 1).

The size of the polycrystalline particles is depicted by the SEM image (Figure 4). The particle sizes were estimated to be 240 nm. Most metals, including Ag, have FCC structures and grow from nucleation into twinned and multiply twinned particles with surfaces bordered by the lowest-energy facets [49]. AgCl NPs tend to agglomerate due to the high surface energy and high surface tension of the ultrafine NPs, which may account for the observation of some larger NPs. SEM can be used to estimate the particle size and XRD can be used to calculate the crystallite size. The crystallite size is different from the particle size. A particle may be made up of several different crystallites [50]. This is the main reason for the difference in particle sizes measured by SEM and XRD.

**Figure 7:**
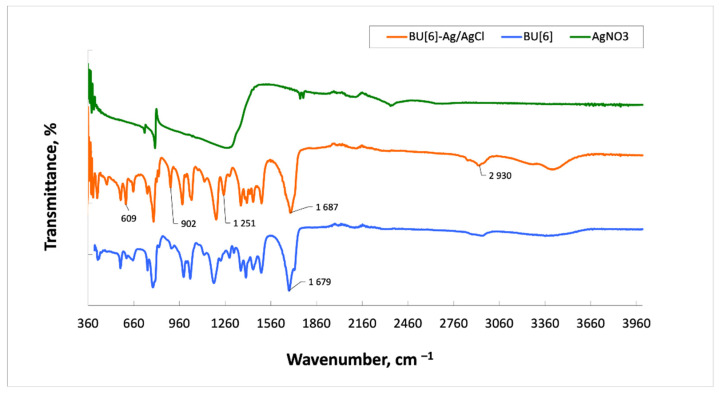
FTIR spectra of BU[6], AgNO_3_, and BU[6]-Ag/AgCl.

Figure 7 shows the FTIR spectra of BU[6], AgNO_3_, and BU[6]-Ag/AgCl. The IR spectrum of BU[6]-Ag/AgCl NPs exhibits a new absorption peak at 1251 cm^−1^, indicating the formation of van der Waals interactions between silver and the carbonyl groups of bambusuril[6]. Additionally, the peak at 609 cm^−1^ indicates the binding of Ag to oxygen C=O bambusuril[6]. As observed from the spectra, the position of the characteristic carbonyl peak of bambusuril[6] shifted to the high-frequency region in the BU[6]-Ag/AgCl NPs spectrum, from 1679 to 1687 cm^−1^, which indicates a supramolecular interaction between carbonyl groups and the surface of NPs.

**Figure 8:**
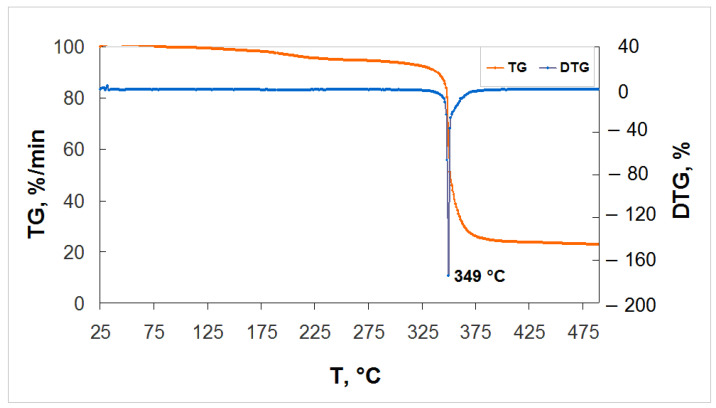
Thermal analysis of BU[6]-Ag/AgCl.

The thermograms of bambusuril[6] and BU[6]–AgCl show a loss of mass during thermal decomposition (Figure 8). The thermogram of bambusuril[6] indicates multistage degradation, with the first mass loss resulting from the evaporation of water and the next mass loss occurring due to the melting of bambusuril[6].

**Figure 9:**
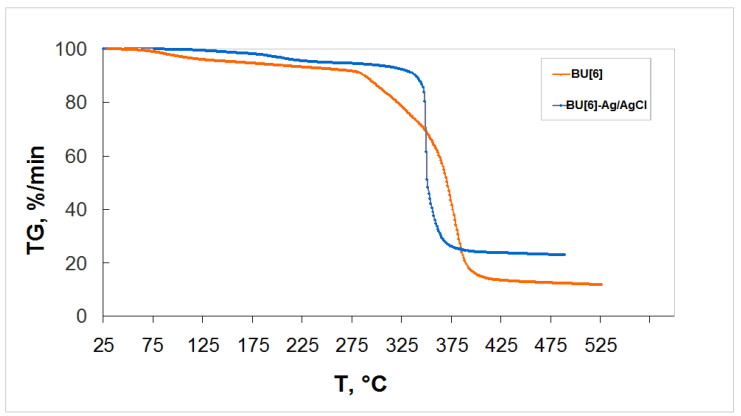
Thermal analysis for BU[6] and BU[6]-Ag/AgCl.

At the end of the experiment, the residue of pure bambusuril[6] at 500 °C was found to represent 10.76% of the total mass. For BU[6]-Ag/AgCl, the first stage of degradation involves the evaporation of water from the structure, followed by the melting of silver nanoparticles in the second stage. Based on these results, the thermal stability of bambusuril[6] changed upon the addition of silver (Figure 9). At 500 °C, the residue of BU[6]-Ag/AgCl NPs was found to be 22.9% of the total mass. Thus, it can be concluded that the silver content in BU[6]-Ag/AgCl NPs is 12.14%.

Strains of *Staphylococcus aureus* (*S. aureus*) and *Escherichia coli* (*E. coli*) were used as test objects to study the effect of BU[6]-Ag/AgCl NPs on Gram-positive and Gram-negative microflora. An 0.9% NaCl solution was added to the medium as a negative control, and bambusuril[6] and BU[6]-Ag/AgCl NPs solutions were added as samples. The concentration of the bacteria used in this measurement was about 1 × 10^8^ cells/mL, and the concentration gradient of BU[6]-Ag/AgCl NPs was from 8 to 1 mg/mL. When the inhibition diameter of pure bambusuril[6] was examined, it was seen that there was no inhibition zone in either type of bacteria. These results showed that pure bambusuril[6] did not have an antimicrobial effect on both bacteria species. It can be seen that BU[6]-Ag/AgCl NPs displayed antimicrobial activity against both *S. aureus* and *E. coli* (Table 2). The zone of inhibition was found to increase in accordance with an increasing concentration of BU[6]-Ag/AgCl NPs. *E. coli* was a bit more sensitive to BU[6]-Ag/AgCl NPs than *S. aureus*, which was shown by the larger zones of inhibition. It was observed that 1 mg/mL BU[6]-Ag/AgCl NPs did not have an antimicrobial effect on either *E. coli* or *S. aureus*. In this study, the MICs of BU[6]-Ag/AgCl NPs against *S. aureus* and *E. coli* were determined by the macrodilution method, and both were found to be effective at 2 mg/mL. One study demonstrated that the MIC of 10 nm silver NPs is a concentration of 1.35 mg/mL against *S. aureus* [51].

It was found that bambusuril[6] reduced the cytotoxicity of porous materials in our previous studies. The surface of titanium nickelide was modified with bambusuril[6] [43]. In vitro tests proved the high biocompatibility and low toxicity of porous TiNi treated with BU[6] under vacuum. The control sample and the sample with the surface modified under vacuum exhibited enhanced surface cytocompatibility. The percentage of live cells MCF-7 in these samples exceeded 90%.

There is no information about the biocompatibility of bambusuril[6]. The biocompatibility of bambusuril[6] was assessed by studying hemolysis (Table 3). The level of hemolysis for bambusuril[6] is 0.3%. It was found that bambusuril[6] does not cause erythrocyte death, since the level of hemolysis of biomaterials in contact with the internal environment of the body does not exceed 5% [52], which indicates the cytocompatibility and non-toxicity of BU[6].

**Figure 10:**
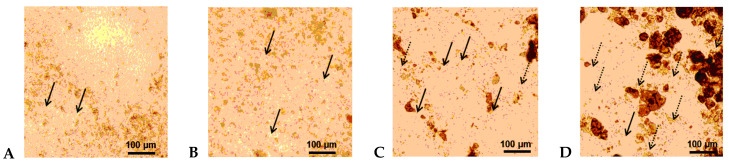
Micrograph of the culture of human mononuclear cells incubated with (**A**,**C**) BU[6] and (**B**,**D**) BU[6]-Ag/AgCl NPs. The incubation durations were 24 h (**A**,**B**) and 144 h (**C**,**D**). A total of 1 mL of cell medium containing cells at a concentration of 1 × 10^6^ cells and 10 µL of the sample suspension (BU[6]-Ag/AgCl NPs; BU[6]) was added to each well of a 24-well plate. Black arrows—live cells, dotted arrows—live cells.

A preliminary investigation was conducted to determine the cytotoxicity of BU[6] and BU[6]-Ag/AgCl NPs on human immune system cells (Table 4). Leukocyte fractions enriched with monocytes were used as a test system. The cytotoxicity of the samples was studied using the MTT test based on the standard for determining the viability of cell cultures (Figure 10) [53]. Cells cultured on a plate without samples were used as a control condition. The differentiation of living and dead cells was carried out visually according to the method proposed by J. Kzhyshkowska [54]. Living cells are lighter than dead cells and have well-defined shapes. The accuracy of this method was confirmed via fluorescence microscopy by staining with DAPI.

A visual evaluation of the mononuclear viability in the presence of the test objects showed that BU[6]-Ag/AgCl NPs had a significant negative effect on the cell viability after 24 h of incubation. A considerable number of dead cells were observed after 144 h, and a large number of cells were identified as necrotic (Figure 10D). In contrast, bambusuril[6] did not have a significant negative effect on the cell viability (Figure 10A). Similar effects were observed after 144 h of incubation (Figure 10B), but they were more pronounced. Differences in the absolute values of cytotoxicity between donors were due to the differences in the mononuclear content in the blood of donors.

**Figure 11:**
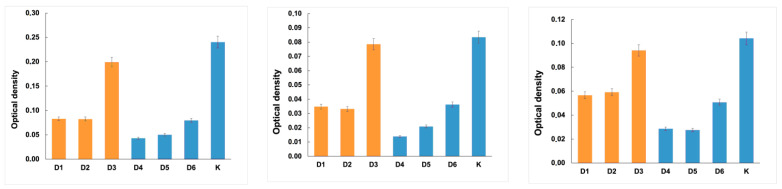
MTT test used for evaluating the cell viability levels of different donors after the incubation of BU[6] and BU[6]-Ag/AgCl NPs for 144 h.

The results of the MTT test for evaluating the cell viability are consistent with the results of the visual evaluation. D1, D4, D5, and D6 had negative impacts on the cells (*p* < 0.05), while the level of mononuclear viability in the presence of sample D3 did not differ from the control sample (*p* > 0.05) (Figure 11). Bambusuril[6], at a concentration 2.5 mg/mL, did not induce mononuclear cell death. The toxicity of bambusuril[6] at a concentration of 10 mg/mL could be attributed to its ability to bind to mononuclear cells. It is known that cucurbituril[7] can form complexes with amino acids, peptides, and proteins [55,56,57]. According to data in the literature, CB[7] can bind to albumin [58,59]. Concentrations of bambusuril[6] higher than 5 mg/mL are not required for medicinal purposes.

There is research in the article of Mitzi J. Ramírez-Hernández regarding the cytotoxic selectivity on cancer cells with biogenically synthesized Ag/AgCl NPs [12]. Systems with Ag/AgCl were tested in mononuclear cells, particularly in monocytes. It was found that NPs were also cytotoxic to monocytes at a concentration of 25 μg/mL. In fact, their half maximal inhibitory concentration (IC50) was lower than that of MCF-7 cells, being 13 and 12 μg/mL, respectively. Interestingly, an unexpected result was that, for concentrations above 35 μg/mL, especially at 50 μg/mL, the cytotoxic effect of NPs was more pronounced on cancer cells than on monocytes [12]. Therefore, the increased cytotoxicity observed with the stabilization of silver NPs by bambusuril[6] against mononuclear cells in vitro suggests that these NPs may possess inherent properties for the effective interaction and eradication of cancer cells. However, these data have never been reported before, so the specific mechanism underlying this process remains unexplored and further investigation is warranted.

## 3. Materials and Methods

Glyoxal was purchased from Novochem (Tomsk, Russia), and silver nitrate was purchased from Reachim (Sverdlovsk, Russia). All other chemicals were purchased from Merck/Sigma–Aldrich (Darmstadt, Germany).

### 3.1. Bambusuril[6] Synthesis

BU[6] was synthesized by the condensation of 2,4-dimethylglycoluryl with formaldehyde in 5.4M HCl, with a yield of 30% [35]. ^1^H NMR (400 MHz, DMSO-d6/CHCl_3_ (1:1), 30 °C, TMS), ppm: 5.29 (s, 12H), 5.06 (s, 12H), 2.51 (s, 36H). ^13^C NMR (100.63 MHz, [D6]DMSO/CDCl_3_ (1:1), 30 °C, TMS), ppm: 159.32, 158.45, 67.82, 48.78, 31.06.

### 3.2. Synthesis of 4,5-Dihydroxy Imidazolidin-2-One

The 4,5-dihydroxy imidazolidin-2-one was synthesized by mixing 50 g of urea with 107 mL of 40% glyoxal, followed by the gradual addition of NaOH until reaching pH 6–7. Once the pH was stabilized, the mixture was heated to 45 °C for 6–7 h. Subsequently, the mixture was cooled, the pH was adjusted to 8, and the mixture was left in a refrigerator at 5 °C for 2 days for crystal formation. The obtained DHI exhibited a melting point range of 157–160 °C. The ^1^H NMR (DMSO-d6 (1:1), TMS) spectrum showed peaks at 7.12 ppm (doublet, 2H), 5.98 ppm (doublet, 2H), and 4.61 ppm (doublet, 2H), while the ^13^C NMR (DMSO-d6 (1:1), TMS) spectrum displayed peaks at 161.01 ppm (C=O) and 84.24 ppm (C-H).

### 3.3. Synthesis of 2,4-Dimethylglycoluril

A total of 20 g of DHI was dissolved in 45 mL of distilled water, followed by the addition of 23 g of dimethylurea. Concentrated sulfuric acid was added gradually to adjust the pH to 2–3. The solution was heated to 85 °C for 2 h, and water was evaporated to yield a precipitate. The obtained precipitate was washed with ethanol. The resulting 2,4-dimethylglycoluril exhibited a melting point of 260 °C. The ^1^H NMR (DMSO-d6 (1:1), TMS) spectrum showed peaks at 7.53 ppm (complex, 2H), 5.12 ppm (complex, 2H), and 2.83 ppm (complex, 6H), while the ^13^C NMR (DMSO-d6 (1:1), TMS) spectrum displayed peaks at 161.56 ppm (C=O), 158.22 ppm (-C=O), 67.67 ppm (C-H), and 28.22 ppm (-CH_3_).

### 3.4. Synthesis of BU[6]-Ag/AgCl NPs

A total of 1.13 g of bambusuril[6] was dissolved in a mixture of solvents consisting of 400 mL of DMSO/CHCl_3_ (1:1). Then, 0.53 g of silver nitrate was added to the solution. The mixture was stirred for 2 h at room temperature with a stirring rate of 150 rpm. The addition of water resulted in the formation of a dark gray precipitate between the water and the organic solvent layers.

### 3.5. Infrared Spectroscopy

Infrared (IR) spectra were recorded using an Agilent Cary 630 Fourier transform infrared spectrometer (Agilent Technologies, Santa Clara, CA, USA) with the attenuated total reflection (ATR) technique (diamond crystal) in the wave number range of 4000–400 cm^−1^.

### 3.6. NMR Spectroscopy

The NMR analysis was conducted using a Bruker AVANCE 400 III HD NMR spectrometer (Bruker, Billerica, MA, USA). One-dimensional spectra were recorded for the 1H (at a frequency of 400.17 MHz) and ^13^C (at a frequency of 100.63 MHz) nuclei to confirm the structure. The solvents used were dimethyl sulfoxide (DMSO D-6) with 99.9% atom D and heavy water (D_2_O).

### 3.7. Thermogravimetric Analysis (TGA)

TGA was conducted using TG-DTA Instruments NETZSCH STA 449F1 (NETZSCH, Selb, Germany). A sample weighing approximately 5 mg was measured and heated from room temperature to 600 °C with a heating rate of 10 °C/min under a nitrogen flow rate of 20 mL/min.

### 3.8. Antibacterial Analysis

The *S. aureus* (ATCC 6538D-5) and *E. coli* (ATCC 25922) strains were used as test objects. The antibacterial activity was determined using the agar diffusion test. Serial two-fold dilutions of BU[6]-Ag/AgCl NPs at concentrations ranging from 8 mg/mL to 1 mg/mL were used to determine the MIC. A test strain was inoculated by the lawn method for each Petri dish with 15 mL of agar medium (0.1 mL of cell suspension at a concentration of 1 × 10^8^ cells/mL, 0.5 McFarland’s standard) from a pure mother culture. Then, a well with a diameter of 7 mm was made in the center of the dish using a sterile cork borer over the entire thickness of the agar layer. A total of 0.1 mL of the sample solution was introduced into the well. After incubation, the zone of bacterial growth inhibition was measured with an accuracy level of 0.1 mm. The MIC endpoint was the lowest concentration of silver NPs for which no visible growth was seen in the tubes.

### 3.9. MTT Test

The MMT test involved the extraction of cells from the leukocyte–platelet layer of a human using the methodology presented by Kzhyshkovskaya Y.G. with modifications by the author [54]. The difference between the used technique and the presented one is that, in the modified version of the technique, the magnetic cell sorting step is omitted, which allows for the exctraction of all mononuclear cells. This makes it possible to assess the overall cytotoxicity of the samples towards immune system cells. Since the samples used in the study were poorly soluble in water, a suspension of the study objects was prepared in a PBS solution at the concentrations indicated in Table 1 for the ex-tempore experiment. After the cells had been extracted, 1 mL of the cell medium containing cells at a concentration of 1 × 10^6^ cells and 10 µL of the sample suspension were added to each well of a 24-well plate. Before each addition of the sample suspension, it was carefully resuspended. The incubation process was carried out at a temperature of 37 °C and 7.5% CO_2_ for 144 h. Cells cultivated on plastic without samples were used as positive controls. The level of cytotoxicity was assessed on mononuclear cells extracted from the blood of three donors—one male (A) and two females (B and C). After incubation, the condition of the cells was visually assessed, and cytotoxicity was assessed using the MTT test method. The optical density was measured using an automatic plate microreader (Tecan Infinite F50, Tecan, Austria) at wavelengths of 560 and 620 nm.

### 3.10. Hemolysis

Healthy donor blood containing sodium citrate (3.8 wt.%) was diluted in a ratio of 9:1 with normal saline (4:5 ratio by volume). The erythrocyte hemolysis test shows the interaction of the entire surface of the biomaterial with blood cells, which are necessary for oxygen transfer by the blood to tissue cells and promote oxidative processes. Samples were dipped into a standard tube containing 10 mL of normal saline that had been previously incubated at 37 °C for 30 min. Next, 0.2 mL of diluted blood was added to the standard tube, and the mixtures were incubated for 60 min at 37 °C. Similarly, normal saline solution was used as a negative control, and deionized water was used as a positive control. After that, all of the tubes were centrifuged for 5 min at 3000 rpm, and the supernatant was carefully removed and transferred to a cuvette for spectroscopic analysis at 545 nm. In addition, hemolysis was calculated using a Uniplan ultraviolet spectrophotometer (Pikon, Moscow, Russia). The hemolysis percent is the average of three replicates, which was calculated as follows:

$$H,\%=\frac{\left(\mathrm{OD}\left(\mathrm{test}\mathrm{sample}\right)-\mathrm{OD}(\mathrm{negative}\mathrm{control}\right)}{\mathrm{OD}\left(\mathrm{positive}\mathrm{control}\right)-\mathrm{OD}(\mathrm{negative}\mathrm{control})}*100\%$$

H, % = percentage of hemolysis

OD(test sample) = absorbance of sample

OD(negative control) = absorbance of negative control with erythrocytes OD(positive control) = absorbance of positive control

### 3.11. XRD

The crystal structure of the solid samples was determined using the X-ray diffraction (CRD) analysis performed on a Shimadzu XRD 6000 diffractometer (Shimadzu, Kyoto, Japan) with Cu K*α* radiation. The data were collected in the angular range of 5° < 2θ > 50° at a scanning rate of 20 deg/min.

### 3.12. X-ray Crystallography

The structural characterization of bambusuril[6] was carried out using a SmartLab SE X-ray diffractometer (Rigaku, Japan). The X-ray source employed copper (Cu) radiation with a power of 2.2 kW. The diffractometer had a vertical goniometer in the Theta–Theta geometry configuration with a measurement diameter of 600 mm. The angular range covered in the analysis was from 10 to 160°.

### 3.13. SEM

The samples structures were studied by scanning electron microscopy (SEM, VEGA 3 SBH, Tescan, Brno, Czech Republic). Energy dispersive X-ray spectroscopy (Oxford Instruments, Abingdon, UK) was used for the elemental analysis.

## 4. Conclusions

This paper presents a simple and effective method for synthesizing silver NPs based on the macrocycle–silver system. Previously, there were no known instances of using bambusuril[6] to obtain silver NPs without the use of conventional reducing agents or external energy sources. The introduction of silver into bambusuril[6] leads to the formation of BU[6]-Ag/AgCl NPs, which exhibit a characteristic surface plasmon resonance peak centered at 430 nm in the UV-visible range. When the SEM images were examined, it was seen that the synthesized BU[6]-Ag/AgCl NPs were distributed with homogeneous sizes, and the synthesized AgNPs were mostly spherical and cubic. The EDS spectra of BU[6]-Ag/AgCl NPs showed the presence of Ag, Cl, and all expected elements. The introduction of Ag^+^ into bambusuril[6] led to the formation of silver NPs with a yield of 30% by mass compared to the theoretical value. Silver NPs stabilized by bambusuril[6] demonstrated high antibacterial activity against *S. aureus* and *E. coli*. The zone of inhibition was found to increase in accordance with increasing concentrations of BU[6]-Ag/AgCl NPs. *E. coli* was a bit more sensitive to BU[6]-Ag/AgCl NPs than *S. aureus*, as shown by the larger zones of inhibition. In this study, the MICs of BU[6]-Ag/AgCl NPs against *S. aureus* and *E. coli* were determined by the macrodilution method, and both were found to be effective at 2 mg/mL. The results of the MTT test for evaluating the cell viability level of mononuclear cells from different donors after incubation for 144 h with BU[6] and BU[6]-Ag/AgCl NPs are consistent with the results of the visual evaluation. It was found that BU[6] had a negative effect on mononuclear cells at concentrations above 10 mg/mL, while the level of mononuclear viability in the presence of BU[6] at a concentration of 2.5 mg/mL did not differ from that of the control sample. BU[6]-Ag/AgCl NPS was shown to induce high toxicity levels for mononuclear cells at all concentrations. The toxicity of bambusuril[6] at concentrations above 10 mg/mL may be associated with its ability to bind to mononuclear immune cells, similar to cucurbituril[7]. Additionally, it should be noted that BU[6]-Ag/AgCl NPs could possess the ability to bind to cancer cells and provoke their demise, further contributing to their anticancer potential. However, these data have never been reported before, so the specific mechanism underlying this process remains unexplored and further investigation is warranted.

## Figures and Tables

**Table 1:** Data showing the crystallite diameter size of the BU[6]-Ag/AgCl NPs.

Miller Indices (hkl)	2*θ* of the Intense Peak (°)	*θ* of the Intense Peak (Radian)	FWHM, *β* (°)	FWHM, *β* (Radian)	Crystallite Diameter, D (nm)
111 (AgCl)	27.71	0.241077	0.25	0.004363	31.40
200 (AgCl)	32.11	0.283692	0.41	0.007156	19.14
220 (AgCl)	46.33	0.409326	0.35	0.006109	22.43
311 (AgCl)	54.93	0.485307	0.25	0.004363	31.40
222 (AgCl)	57.6	0.508896	0.35	0.006109	22.43
111 (Ag)	38.13	0.336879	0.45	0.007854	17.44

**Table 2:** Antimicrobial results of the samples.

Samples	Inhibition Zone of *E. coli* (mm)	Inhibition Zone of *S. aureus* (mm)
Negative control (NaCl)	0	0
10 mg/mL Bambusuril[6]	0	0
1 mg/mL BU[6]-Ag/AgCl NPs	0	0
2 mg/mL BU[6]-Ag/AgCl NPs	12.4	12.1
4 mg/mL BU[6]-Ag/AgCl NPs	13.9	13.4
8 mg/mL BU[6]-Ag/AgCl NPs	17.5	17.4
MIC	2 mg/mL	

**Table 3:** Hemolysis of BU[6].

Samples	Hemolysis, %
BU[6]	0.3
CTRL (plasma)	0

**Table 4:** Investigated materials and abbreviations.

Samples	Name	Concentration, mg/mL
BU[6]	D1	10
BU[6]	D2	5
BU[6]	D3	2.5
BU[6]-Ag/AgCl	D4	10
BU[6]-Ag/AgCl	D5	5
BU[6]-Ag/AgCl	D6	2.5

## Data Availability

The data presented in this study are available in this article.
